# Feasibility and acceptability of undertaking postmortem studies for tuberculosis medical research in a low income country

**DOI:** 10.3389/fimmu.2023.1264351

**Published:** 2023-12-06

**Authors:** Gift Ahimbisibwe, Marjorie Nakibuule, Marvin Martin Ssejoba, David Oyamo, Rose Mulwana, Josephine Nabulime, Febronius Babirye, Musana Abdusalaamu Kizito, Hervé Monka Lekuya, Akello Suzan Adakun, Daisy Nalumansi, Stella Muryasingura, Robert Lukande, Andrew Kyazze, Joseph Baruch Baluku, Irene Andia Biraro, Stephen Cose

**Affiliations:** ^1^ Medical Research Council (MRC)/Uganda Virus Research Institute (UVRI) and London School of Hygiene and Tropical Medicine (LSHTM) Uganda Research Unit, Entebbe, Uganda; ^2^ Mulago National Referral Hospital, Kampala, Uganda; ^3^ Department of Pathology, Makerere University, Kampala, Uganda; ^4^ Department of Surgery, Makerere University, Kampala, Uganda; ^5^ Division of Pulmonology, Kiruddu National Referral Hospital, Kampala, Uganda; ^6^ Department of Internal Medicine, Makerere University, Kampala, Uganda; ^7^ Clinical Research Department, London School of Hygiene and Tropical Medicine, London, United Kingdom

**Keywords:** postmortem studies, feasibility & acceptability, tuberculosis, tissues, T cells

## Abstract

**Introduction:**

If we are to break new ground in difficult-to-treat or difficult-to-vaccinate diseases (such as HIV, malaria, or tuberculosis), we must have a better understanding of the immune system at the site of infection in humans. For tuberculosis (TB), the initial site of infection is the lungs, but obtaining lung tissues from subjects suffering from TB has been limited to bronchoalveolar lavage (BAL) or sputum sampling, or surgical resection of diseased lung tissue.

**Methods:**

We examined the feasibility of undertaking a postmortem study for human tuberculosis research at Mulago National Referral Hospital in Kampala, Uganda.

**Results:**

Postmortem studies give us an opportunity to compare TB-involved and -uninvolved sites, for both diseased and non-diseased individuals. We report good acceptability of the next-of-kin to consent for their relative’s tissue to be used for medical research; that postmortem and tissue processing can be undertaken within 8 hours following death; and that immune cells remain viable and functional up to 14 hours after death.

**Discussion:**

Postmortem procedures remain a valuable and essential tool both to establish cause of death, and to advance our medical and scientific understanding of infectious diseases.

## Introduction

There is no question that the SARS-CoV-2 (COVID-19) pandemic has reversed many of the gains made in fighting the effort to control the infection and spread of *Mycobacterium tuberculosis (M.tb)*, the causative agent of tuberculosis (TB), and has set back the fight against TB by years (1). Nevertheless, we cannot blame our lack of success in controlling TB on COVID-19 alone. We have lived with TB long enough not to have it as the leading cause of death from a single infectious disease prior to SARS-CoV-2, and by all accounts it is set to regain its number one spot again (2). The major challenges to realizing the global 2030 End TB reduction targets include the lack of efficient TB vaccines, diagnostic challenges, drug resistance, and inaccessibility to effective chemotherapy (2, 3).

The best intervention against TB, or in fact any infectious disease, is one that breaks the transmission cycle. Lessons from other infectious diseases that have now been eradicated, such as smallpox and rinderpest, have shown that vaccination is the best way to break transmission; polio is almost eradicated as well due to the WHO’s determined vaccination strategy (4). TB vaccination has been extensively taken up by most TB-endemic countries. However, it is well-known that BCG, currently the only licensed TB vaccine, is not effective against adult pulmonary TB, the most transmissible form of the disease in endemic countries (5). We need to develop more effective TB vaccines and develop shorter drug regimens, amongst a plethora of other interventions (2). The last few years have witnessed a reawakening of novel TB vaccine approaches. There is hope in some current trial vaccines (M72, BCG REVAC, VPM1002, H56/IC31), but tertiary trial results will not be out for several years (6, 7). In the meantime, we need vaccine strategies based on deeper insights into the immunity underlying TB, and not just in blood, but most importantly, at the site of infection in humans.

This is not to say that we have little idea of tissue-specific cell subsets in human tissue; tissue-resident immune cells have been successfully isolated from the lungs of organ donors (8, 9), and include subsets such as tissue-resident memory (TRM) T cells (10), macrophages (11), NK and innate lymphoid cell (ILC) subsets (12–14), mucosal-associated invariant T (MAIT) cells (15, 16) and γδ T cells (17). But only a few studies have looked at these subsets in the lungs of TB-diseased individuals (17–19), and none in otherwise healthy individuals with a latent TB infection (LTBI). Studies have been largely restricted to relatively accessible sites such as bronchoalveolar lavage (BAL) (20–22), the pleural fluid (23–25), or occasionally pleural or lymph node biopsies when clinically indicated (26). New techniques such as Positron Emission Tomography – Computed Tomography (PET-CT) can help us to understand the resolution or progression of disease (27–31), but cannot assess specific cellular functions. We therefore have only a superficial knowledge of how the immune system responds at the main site of TB infection and disease in humans – the lungs.

To address this, we conducted a postmortem study to determine whether we could use immune cells isolated from tissues taken postmortem for medical research. We show that we were able to get consent from the next of kin(s) (NoKs), perform a full postmortem, and collect and process tissues with little loss in cell viability or function, up to 14 hours post-death.

## Materials and methods

### Ethics

This study obtained ethical approval from five ethics bodies: the Makerere University School of Biomedical Sciences Research & Ethics Committee (SBS-REC-721), the Mulago National Referral Hospital Ethics Committee (MHREC 1849), the Kiruddu National Referral Hospital School of Biochemical Research and Ethics Committee (CRD/ADMIN/120/1), the Uganda National Council of Science and Technology Ethics Committee (HS703ES), and the London School of Hygiene and Tropical Medicine Ethics Committee (22922).

### Consent

Trained grief counselors were employed to sensitively obtain informed consent from the next-of-kin (NoK) as described by Uganda Law. The NoK gave consent for a full postmortem, for donation of tissues for medical research and future use, and for access to previous medical records. A case record form was used by the counselors to collect reasons for consent or decline from the NoK.

### Participants

We recruited deceased subjects from Mulago National Referral Hospital in Kampala, Uganda, between January 2021 and June 2022. Only subjects above 18 years of age whose NoK consented to the study were recruited into the study. Active TB subjects were recruited from Wards 4, 5, and 6 of Mulago National Referral Hospital (MNRH), as well as the Infectious Diseases Ward. We used road traffic accident victims as our non-TB subjects, who were recruited from the Accident and Emergency Unit of MNRH (referred to as the Surgical Emergency Unit, SEU). Recruited subjects from this Unit were otherwise healthy at the time of death, with no obvious underlying morbidity or chest involvement at the time of death. Patient demographics are described in **Table 1**.

**Table 1:** Patient demographics.

	SEU^a^	TB^b^
Gender		
Male	37	20
Female	10	13
Age Range		
18-35	26 (26.3)^c^	13 (28.6)
36-55	12 (41.7)	15 (43.8)
≥56	9 (69.4)	5 (67)
HIV status		
Positive	6	23
Negative	41	10
Cause of death		
Trauma
	
Head trauma	35	
Abdominal	8	
Gunshot	1	
Non trauma
	
Intestinal obstruction	2	
Duodenal ulcers	1	
TB Cases
	
Pulmonary TB		12
Extrapulmonary TB		13
HIV associated		8
**Total**	47	33

Data shows collated patient demographics across the whole study.

a Surgical Emergency Unit.

b Tuberculosis Wards.

c Parentheses represent average age.

### Tissues and sample collection

A full postmortem was performed by the study pathologist on all study subjects to establish the cause of death and identify any underlying conditions that may have been missed during routine clinical and laboratory examination. Tissue samples, including lung, lymph nodes draining the lung (Hilar Lymph Nodes - HLNs), spleen, distal lymph nodes (Iliac and inguinal lymph nodes – DLNs), and blood were collected. The solid tissues were placed in 50ml tubes with 20% FBS in RPMI medium. Bronchoalveolar lavage (BAL) washing of both the left and right lungs was performed with PBS. Arterial blood was collected from the carotid artery into heparin tubes; arteries were considered instead of veins due to regular venous collapse after death. Sample tubes were tightly capped, placed in a rack in a sealable cool box, and transported to the BSL3 laboratory at the MRC/UVRI and LSHTM Uganda Research Unit at room temperature. All sample processing was performed under BSL3 conditions.

### PBMC isolation

Heparinized blood was diluted with RPMI media and layered manually on FicollPaque PLUS media. PBMCs were then isolated by density centrifugation. The buffy coat was harvested and centrifuged to obtain a cell pellet. Red blood cells in the pellet were lysed, then the cells were washed and counted on an automated cell counter (TC20; Biorad). 

### Cell isolation from tissues

#### Lung

Cells were obtained from lung tissue by enzymatic digestion using collagenase D (1mg/ml) and DNase I (1g/ml) (hereafter referred to as “the enzyme mixture”) and physical disintegration using the gentleMACS Octo Dissociator (Milteyni Biotech) as described previously (8). Briefly, the lungs were cut into small pieces using fine scissors and forceps in a sterile petri dish, placed in gentleMACS purple C tubes with the enzyme mixture, loaded on the gentleMACS Octo Dissociator, and run using Lung Program 1. The tubes were then incubated in a CO_2_ incubator for 25 minutes and then run using Lung Program 2. 1ml of smashed tissue was taken off to perform MGIT culture. The sample was then filtered through a 70 µm filter followed by a 40 µm filter and centrifuged at 600 rcf for 5 minutes to obtain a cell pellet. Residual red blood cells were lysed using ACK lysis buffer, leaving behind a pure black cell pellet. We assume the cell pellet from the draining lungs was black due to a lifetime of exposure to carbon residues as a result of inhalation of carbon fumes from vehicles, firewood, and charcoal smoke (32). The cells were washed in RPMI and counted using an automated TC20 cell counter (Biorad).

#### Lymph nodes

Lymph nodes were first cleaned by teasing the lymph node tissue from surrounding fat using forceps and scissors, cut into small pieces using fine scissors and forceps in a sterile petri dish, and mixed with RPMI media. The resultant mixture was then filtered through a 70 µm filter followed by a 40 µm filter, then centrifuged at 600 rcf for 5 minutes to obtain a cell pellet. Red blood cells were lysed using ACK lysis buffer. The resultant pellet was washed with RPMI and counted using an automated TC20 cell counter.

#### Spleen

Spleen sections were chopped into small pieces using fine scissors and forceps in a sterile petri dish and mixed with RPMI media. The resultant mixture was then filtered through a 70 µm filter and centrifuged at 600 rcf for 5 minutes to obtain a cell pellet. The pellet was then reconstituted with RPMI and layered manually onto FicollPaque PLUS media. Spleen mononuclear cells were then obtained by density centrifugation. The buffy coat was harvested and centrifuged to obtain a cell pellet. Residual red blood cells in the pellet were lysed using ACK lysis buffer, and the cells were then washed and counted using an automated TC20 cell counter.

#### Bronchoalveolar lavage fluid (BAL)

BAL was collected in 50ml PBS, using a standard BAL procedure, but washing both lungs and deeper airways than would normally be performed for a living subject. BAL fluid was filtered through a 70 µm filter followed by a 40 µm filter, then centrifuged at 600 rcf for 5 minutes to obtain a cell pellet. Red blood cells were lysed using ACK lysis buffer. The resultant pellet was washed with RPMI and counted using an automated TC20 cell counter.

### T-SPOT^®^.*TB* assay

This assay was performed using the T-SPOT^®^.*TB* kit (TB.300, Oxford Immunotec) to enumerate individual TB-specific activated effector T cells producing IFNγ. Samples were run in duplicate. The procedure was performed as per insert, except for incubation time (36 hrs) and media (RPMI plus 10% FBS). Extensive testing showed that this time and media yielded the best results from our deceased subjects, above that of the recommended protocol for live venous blood. Resultant spots were read using an ELISPOT reader (AID *i*Spot ELR08IFL), and were interpreted as per insert.

### BACTEC MGIT 960 for recovery of *M.tb*

Tissue or BAL was processed as per the BD BBL MycoPrep decontamination kit (Cat. No. 240862, 240863), with minor modifications. Briefly, tissue or BAL was decontaminated with MycoPrep for 30 minutes while vortexing every 5 minutes. Positive and negative controls of known time to positivity and bacterial load were included to control for the decontamination process. Sterile Phosphate Buffered Saline (PBS) was then added to stop the reaction followed by a 3500g spin for 15 minutes. The sediment was then inoculated into the MGIT tubes with BBL™ MGIT™ PANTA™ Antibiotic Mixture (245114) to minimize contamination and enhance *M.tb* growth. MGIT tubes were then incubated in the BD BACTEC™ MGIT™ Automated Mycobacterial Detection System for 42 days. Positive growths were confirmed by positive Ziehl-Neelsen (ZN) staining and the MPT64 antigen assay. For all of the samples included in this manuscript, no tissue or BAL sample was confirmed as *M.tb* positive amongst the non-TB subjects.

### T cell phenotypic analysis by flow cytometry

**Figure 1:**
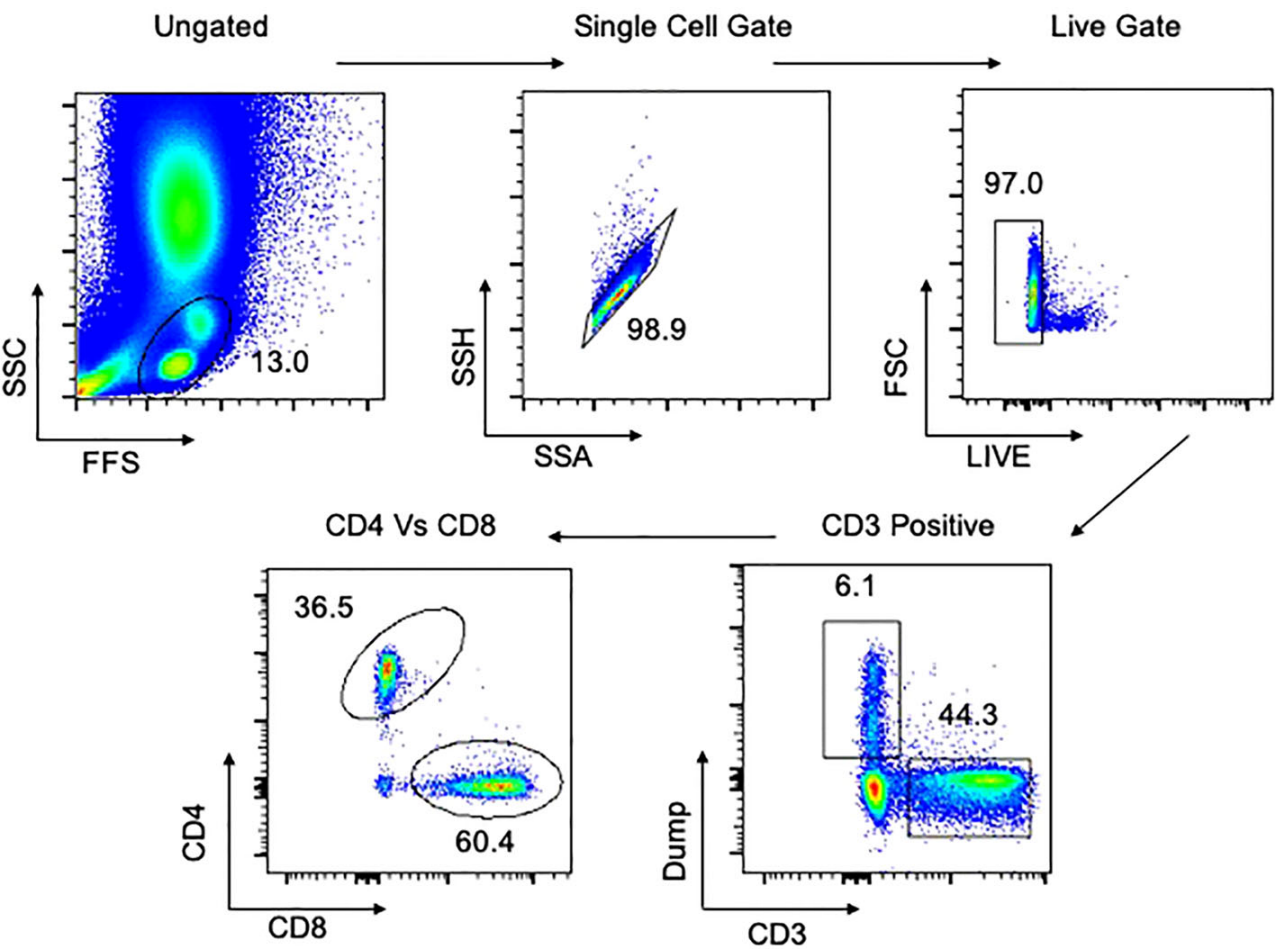
Gating strategy for flow cytometry in PBMCs. Representative plot of total cells from arterial PBMCs taken postmortem. Total cells are first gated by FSC/SSC, then singlets isolated, followed by selection of live cells and then CD3 positive cells. The dump gate, used to make the CD3 gate cleaner, consists of CD19/CD56/CD14. Finally, cells are separated based on CD4 and CD8, followed by specific staining as represented in the Results. Data comes from a 65-year-old male HIV-/TB- subject recruited from the SEU.

**Figure 2:**
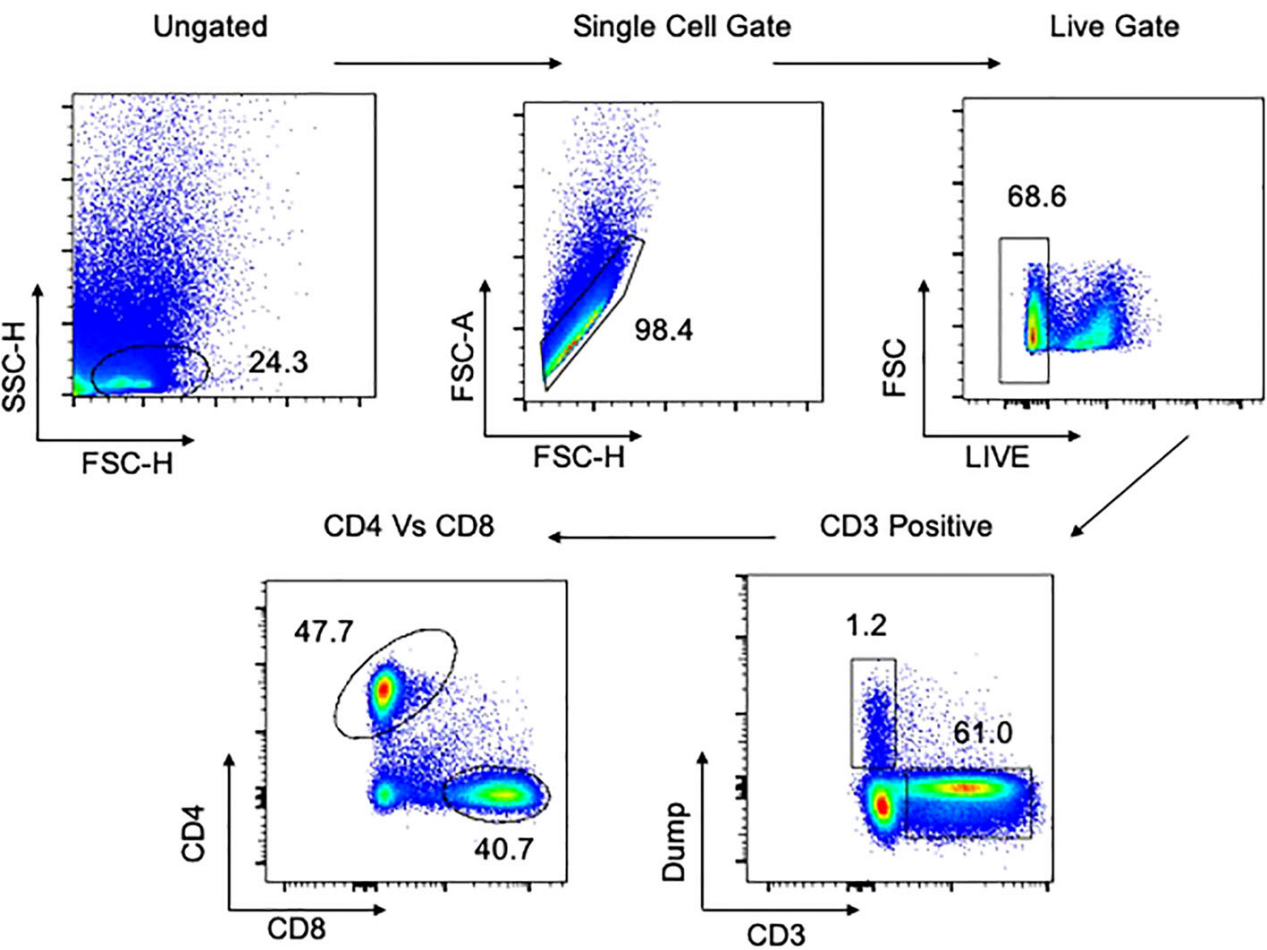
Gating strategy for flow cytometry in tissues. Representative plot of total cells from lung tissue taken postmortem. Total cells are first gated by FSC/SSC, then singlets isolated, followed by selection of live cells and then CD3 positive cells. The dump gate, used to make the CD3 gate cleaner, consists of CD19/CD56/CD14. Finally, cells are separated based on CD4 and CD8, followed by specific staining as represented in the Results. A similar gating strategy was employed for all other tissues. Data comes from a 65-year-old male HIV-/TB- subject recruited from the SEU.

A 29-color antibody T and B cell panel was used to phenotype cells isolated from each of the tissues. The gating strategy for PBMCs and tissues are shown in **Figures 1**, **2**, respectively. Antibodies used were CD3 (PACIFIC BLUE HIT3a BioLegend), CD38 (BV510 HIT2 Biolegend), CCR7 (BV605 G043H7 Biolegend), PD-1 (BV650 NAT105 Biolegend), CD69 (BV711 FN50 Biolegend), CD28 (BV750 CD28.2 Biolegend), CD27 (BV785 O323 Biolegend) CD8 (SPARK BLUE 550SK1 Biolegend) FCLR4 (PE413D12 Biolegend), CD127 (SPARK YG581 A019B5 Biolegend) CD4 (PECY5 RPA-T4 Biolegend), CD57 (PECY7 HNK-1 Biolegend), IgG (APC M1310G05 Biolegend), KLRG1 (ALEXAFLUOR 647 SA231A2 Biolegend), CD21 (ALEXAFLUOR 700 Bu32 Biolegend) HLADR (APC-FIRE 810 I243 Biolegend), LIVE/DEAD (ZOMBIE UV Biolegend) CD103 (BUV395 Ber ACT8 BD), CD25 (BUV496 M-A251 BD), CD196 (BUV563 11A9 BD), CD278 (BUV661 DX29 BD), IgM (BUV737 UCH-B1 BD), CD45RA (BUV805 HI100 BD), CD5 (BV421 UCHT2 BioLegend), CD24 (FITC eBioSN3 eBioscience), CD183 (BB700 1C6/CXCR3 BD), CD10 (PerCP efluor710 SN5c Life technologies), IgD (PE-Dazzle594 IA6-2 BD), CD19 (APC-H7 SJ25C1 BD).

Viability staining was performed first with the fixable viability dye, zombie UV, for 20 minutes in the dark at room temperature. Cells were washed and resuspended in 100 µl of surface antibody cocktail for 20 minutes in the dark at 4^°^C, after which they were washed to remove excess antibody. Cells were acquired using a Cytek Aurora Spectral Analyser. All flow cytometry data was analysed using FlowJo version 10.8.1. Compensation controls to remove spectral overlap and Fluorescence Minus One (FMO) controls were used to establish gates. Representative gating strategies for PBMC and lung samples are shown in **Figures 1**, **2**, respectively.

### Statistical analysis

FlowJo and GraphPad Prism Version 9 software were used for graphical representation and statistical analysis.

## Results

To determine whether tissue-specific immunology studies could be performed on tissues donated for medical research following death, we assessed two essential elements: 1) could we get consent from the NoK, and if so, 2) how viable would cells be following death and the postmortem process.

We found that we could get consent from the NoK with very high acceptability rates at the SEU (94% of those approached; **Table 2**), and with more modest rates on the TB and ID Wards (35% of those approached; **Table 2**). The latter consent rate was in line with a previous study assessing consent across all Wards at MNRH (33). The high rate of consent at the SEU was a surprise, as we had assumed a sudden, unexpected death might mean that the NoK would be less willing to consent, whereas the consent rate on the TB and ID Wards might be higher because the patient and family have usually built a rapport with hospital staff, and may therefore be more keen to know the actual cause of death of their relative. Most NoKs believed that understanding the reason for a loved one’s sudden death would help them to better come to terms with their loss; the low consent rate on the TB and ID Wards was mainly attributed to religious norms that do not allow a postmortem to be undertaken (**Table 2**).

**Table 2:** Reasons for consent and decline.

Numbers	SEU^a^	TB^b^
Met study criteria	50	94
Consented	47	33
Declined	3	61
Consent rate	94%	35%

Reasons for Consent	SEU^a^	TB^b^
Gender		
Male	13	10
Female	34	23
Knowing the cause of death will help prevent more deaths	5	10
It is important in settling NSSF, deceased’s bank payments	8	8
Understanding the reason for a loved one's death helps them come to terms with their loss	17	9
It sounds like the right thing to do	9	1
It is a way of saving future lives	4	5

Reasons for Decline	SEU^a^	TB^b^
Gender		
Male	1	27
Female	2	34
Reasons for decline		
Religious norms	1	16
Cultural norms	1	12
Fear of body disorientation	1	8
Process will delay funeral arrangements		10
PM not necessary		2
Lack of authority		3
Not sure deceased would approve		4
No reason		6

Whilst approaching families for consent to the study, we asked a short questionnaire on why they did or didn’t accept for their deceased family member to be a part of the study, and for the use of their tissues for medical research. Data shows the responses given by the families that agreed to answer the questionnaire.

a Surgical Emergency Unit.

b Tuberculosis Wards.

**Figure 3:**
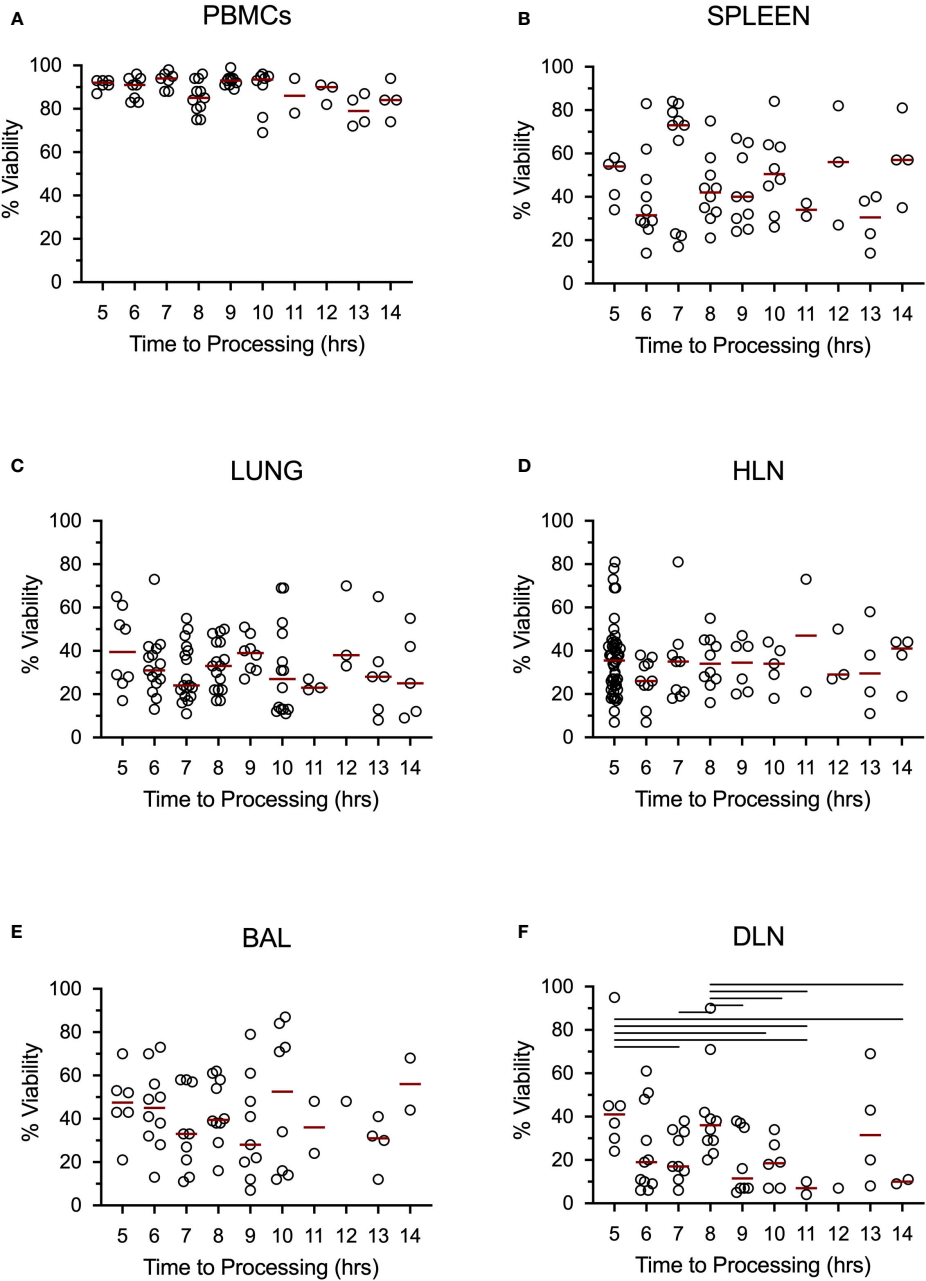
Tissue cells collected postmortem are viable up to 14 hours post-death. Data shows percent viability of isolated single cells from the Blood **(A)**, Spleen **(B)**, Lungs **(C)**, Hilar lung draining lymph nodes **(D)**, Bronchoalveolar lavage **(E)**, and distal, non-draining lymph node (iliac lymph nodes) **(F)**. Bars represent medians and lines above each graph represent a significant difference between the two groups at a significance level of p < 0.05. Data came from all subjects.

After obtaining consent, the body was taken for a full postmortem. Tissues were taken to assess the viability of cells over time, from the time of death through sample processing to cell counting in the laboratory. We found that cells were viable up to 14 hours post-death, but that there was considerable variability in viability depending on the tissue sample (**Figure 3**). Arterial blood maintained a high level of viability out to 14 hours post-death, but other tissues examined were considerably lower; around 40% of the cells isolated were viable. A regression analysis showed no effect of time from death on viability for any tissue, with the exception of PBMCs. For PBMCs, we found a significant decrease of 1.4% viability for every hour following death that the samples were processed. We did not see a difference in cell viability in any tissue between TB and non-TB groups.

**Figure 4:**
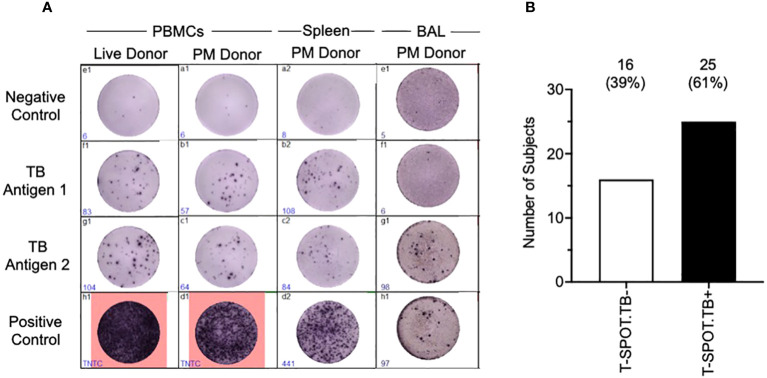
Tissue cells collected postmortem are functional in a T-SPOT^®^.*TB* Assay. T-SPOT^®^.*TB* data **(A)** shows that arterial PBMCs isolated and collected postmortem perform as well as venous blood from a live donor. In addition, the T-SPOT^®^.*TB* assay also works in tissues such as the spleen and BAL. Culture conditions were slightly altered to enable the assay to work, as described in the methods. **(B)** shows latent TB infection (LTBI) data collected from our non-TB arterial blood samples, assayed using the T-SPOT^®^.*TB* assay. Data are from HIV-/TB- people recruited from the SEU.

Having shown that we could isolate viable cells from the organs of deceased subjects, we next tested whether the cells were functional using the T-SPOT^®^.*TB* assay to assess exposure to *M.tb* antigens (ESAT-6 and CFP-10) (**Figure 4**). We found that PBMCs isolated postmortem were just as functional as PBMCs isolated from the venous blood of a living subject (**Figure 4A**). Furthermore, the T-SPOT^®^.*TB* assay worked on spleen and bronchoalveolar lavage (BAL) samples, showing that cells isolated from tissues are not just viable, but are functional as well. In undertaking the T-SPOT^®^.*TB* assay, we were able to identify both T-SPOT^®^.*TB* positive and negative subjects (**Figure 4B**). PBMCs isolated postmortem from HIV-positive blood did not give reliable responses, so HIV-positive participants were excluded from this analysis. We found that 61% (**Figure 4B**) of the subjects tested were T-SPOT^®^.*TB* positive – a finding similar to other studies conducted in the same population (34, 35), and suggesting no overt bias in our non-TB postmortem population.

**Figure 5:**
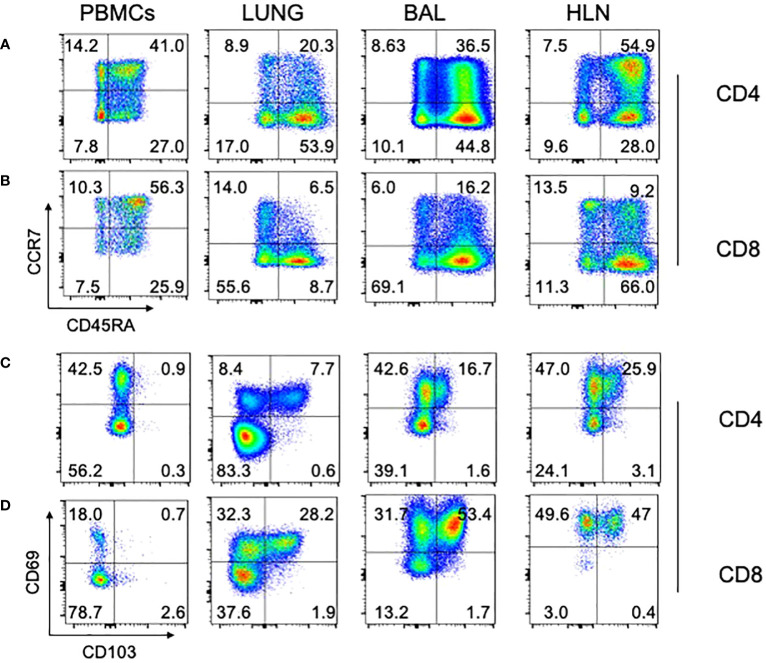
Naïve, memory, and TRM subsets in different human tissues. Standard phenotypical markers (CCR7/CD45RA) were used to differentiate memory subsets within the indicated tissues for CD4 **(A)** and CD8 **(B)** T cells. Tissue-specific memory T cell markers (CD69/CD103) were also used to identify cell subsets within human tissue, again for both CD4 **(C)** and CD8 **(D)**. Note that lung tissue-specific memory T cells (CD69+/CD103+) cells are absent from PBMCs, but present in lung tissue, BAL, and HLN. Representative plots are shown, from a 74 year old HIV-/TB- person recruited from the SEU. BAL, Bronchoalveolar lavage; HLN, draining lung hilar lymph node.

Lastly, we asked whether T cells isolated from different organs showed evidence of classical immunological naïve and memory T cell subsets (**Figures 5A, B**; representative data). In all organs analyzed, both CD4 (**Figure 5A**) and CD8 (**Figure 5B**) T cells had naïve (CCR7^+^CD45RA^+^; T_N_), central memory (CCR7^+^CD45RA^-^; T_CM_), effector memory (CCR7^-^CD45RA^-^; T_EM_) and effector memory RA (CCR7^-^CD45RA^+^; T_EMRA_) subsets, albeit at different frequencies. Additionally, we asked whether Tissue Resident Memory (TRM) T cells, defined by co-expression of CD69 and CD103, were present in the different organs, for both CD4 (**Figure 5C**) and CD8 (**Figure 5D**) T cells. As expected, TRM T cells (CD69^+^CD103^+^) could be identified in the lung tissue (LUNG), bronchoalveolar lavage (BAL), and the lung draining hilar lymph nodes (HLN), but were not found in the blood.

## Discussion

Development of better TB vaccine strategies needs to be based on deeper insights into the immunity underlying TB, and not just in blood, which is commonly undertaken, but perhaps more importantly, at the site of infection. Our knowledge of TB immune responses at the main site of infection, the lungs, is largely derived from animal models and whilst clearly important, few animal models exist that recapitulate the complex natural history of disease progression and reactivation as it exists in humans. Non-human primate (NHP) models are our closest model to human infection and disease, but are limited by cost and exposure to concurrent (or prior) unrelated infections and vaccine responses. Current human lung studies are sampled from patients with severe lung complications at the extreme end of the TB disease spectrum, and often lack normal lung tissue from non-diseased individuals for comparison. Another approach to tackle the burden of disease is to undertake postmortem studies. Postmortem studies allow us to not only compare the immunology within the tissues of people who have died from TB disease from those who have died of other causes, but also allow us to study the immunology of disease at other, non-involved tissues within the same individual. Postmortem studies also allow us to sample the entire population, and study tissue-specific responses in otherwise healthy people at the time of death – road traffic accident victims, for example.

We report that hospital-driven postmortems are feasible in Uganda. Aligned with a previous study (36), we found that consent rates were highly dependent on whether death was sudden or not. The consent rate on the TB and ID Wards was more than two-fold lower than at the SEU, but comparable to what has previously been reported at Mulago National Referral Hospital (33). The reason for this may be that on Wards other than the SEU, patients are admitted and stay for longer periods of time, during which a clinical diagnosis is established, and relatives therefore have no need for a postmortem to be conducted to determine the cause of death. Indeed, the major reason for consent given by the NoKs at the SEU, where the average length of a hospital stay for RTA victims was 10 hours, was that knowing the cause of death helped in coming to terms with their relative’s death.

In this paper, we show that lymphocytes remain viable and functional up to 14 hours post-death. Previous studies have reported good cell viability up to several days following death (37–39), far longer than the time frame of our study. However, few studies have looked at the functionality of cells isolated postmortem (8, 9), even fewer in tissues, and even fewer that are focussed on *M.tb* (17–19). Of note, the T-SPOT^®^.*TB* test that we used for *M.tb*-specific functionality required a change of incubation time from the manufacturer’s recommendation of 24 hours to 36 hours. In addition, changing the media to RPMI from the recommended media (AIMV) yielded more consistent results. It is unclear why these two changes improved the consistency of the T-SPOT^®^.*TB* test in our case.

Finally, we presented a snapshot of T cell subsets in the blood and tissues of a subject from the SEU, showing that 1) classical naïve and memory T cell subsets were present in all organs, but at different frequencies, and 2) TRM T cells could be detected within the tissues and were absent from the blood, as would be expected. This work presents the platform under which we can undertake further research to understand the immune response to TB within different human tissues, and between those individuals who died from TB and those who were otherwise healthy at the time of death, but died from other causes, such as RTA victims.

## Conclusion

This study presented the feasibility of undertaking postmortem studies for medical research. A clear limitation in this kind of hospital-based study is that patient demographics are skewed; this is particularly the case for TB subjects and is a well-recognized epidemiological quirk. In addition, our non-TB subjects were skewed in terms of gender, limiting the overall generalisability of this study; ongoing recruitment of further subjects may help resolve this issue.

These limitations notwithstanding, we have shown that postmortem studies are a feasible approach to studying tissue-specific immune responses to disease in humans. Lymphocytes can be isolated from tissues up to 14 hours after death with no loss of viability. These cells are functional and show tissue-specific surface markers. Different tissues show clear differences in classical T cell memory and TRM subsets. Human postmortem procedures remain a valuable and essential tool to advance our medical and scientific understanding, not just for understanding TB disease and its progression, but for any disease with a tissue-specific tropism. Although largely fallen out of favor in first-world settings, postmortem studies are clearly a valuable tool in our race to understand immune responses at the site of infection, and in the rational design of future tissue-specific vaccines.

## Data availability statement

The raw data supporting the conclusions of this article will be made available by the authors, without undue reservation.

## Ethics statement

The studies involving humans were approved by School of Biological Sciences Research Ethics Committee (SBS REC), Makerere University, Kampala, Uganda Mulago National Referral Hospital Research Ethics Committee (MNRH REC), MNRH, Kampala, Uganda Kiruddu National Referral Hospital Research Ethics Committee (KNRH REC), KNRH, Kampala, Uganda Uganda National Council for Science and Technology (UNCST), Kampala, Uganda London School of Hygiene and Tropical Medicine Research Ethics Committee (LSHTM REC), London, UK. The studies were conducted in accordance with the local legislation and institutional requirements. The participants provided their written informed consent to participate in this study.

## Author contributions

GA: Investigation, Methodology, Writing – original draft, Writing – review & editing, Data curation, Project administration. MN: Data curation, Investigation, Methodology, Writing – review & editing. MM: Investigation, Methodology, Writing – review & editing. DO: Investigation, Writing – review & editing. RM: Investigation, Writing – review & editing. JN: Investigation, Writing – review & editing. FB: Investigation, Writing – review & editing. MK: Investigation, Writing – review & editing. HL: Investigation, Writing – review & editing. AA: Investigation, Writing – review & editing. DN: Investigation, Writing – review & editing. SM: Investigation, Writing – review & editing. RL: Investigation, Writing – review & editing. AK: Investigation, Writing – review & editing. JB: Investigation, Writing – review & editing. IA: Investigation, Writing – review & editing, Conceptualization. SC: Conceptualization, Investigation, Writing – review & editing, Funding acquisition, Methodology, Resources, Supervision, Writing – original draft.
